# Acute Myocardial Infarction Caused by Plaque Erosion After Recovery From COVID-19 Infection Assessed by Multimodality Intracoronary Imaging

**DOI:** 10.7759/cureus.25565

**Published:** 2022-06-01

**Authors:** Kazuki Matsuda, Tomoyo Sugiyama, Masahiro Hoshino, Tsunekazu Kakuta

**Affiliations:** 1 Department of Cardiovascular Medicine, Tsuchiura Kyodo General Hospital, Tsuchiura, JPN

**Keywords:** case report, covid-19, optical coherence tomography, coronary angioscopy, plaque erosion, acute coronary syndrome

## Abstract

We report a case of non-ST-elevation acute myocardial infarction after recovery from COVID-19 infection. An emergency coronary angiography revealed 50% stenosis with thrombotic occlusion in the middle left anterior descending artery. Optical coherence tomography and coronary angioscopy revealed plaque erosion with mixed thrombus on a lipid-rich plaque. This case report may help to understand the underlying mechanisms of cardiac complications following COVID-19 infection.

## Introduction

The novel coronavirus disease 2019 (COVID-19) infection caused by severe acute respiratory syndrome coronavirus 2 (SARS-CoV-2) increases the incidence of thrombotic complications including myocardial infarction [1]. However, the characteristics of culprit plaque in myocardial infarction related to COVID-19 have not been fully evaluated. We present a case of a patient with acute myocardial infarction (AMI) caused by plaque erosion with intracoronary thrombus evaluated by invasive coronary angiography, optical coherence tomography (OCT), and coronary angioscopy (CAS) who had suffered from COVID-19 infection one month earlier.

## Case presentation

A 43-year-old man was transferred to our emergency department due to sudden-onset chest pain. He had a history of hypertension, dyslipidemia, and current smoking. He had suffered from COVID-19 infection one month earlier and recovered after a 10-day-monitored quarantine at home. He had never received the vaccination for COVID-19 prior to the index infection. He had a mild cough and a fever of 37°C during the quarantine period, but he was freed from any respiratory symptoms and fever. The 12-lead electrocardiogram showed sinus rhythm and no significant ST-segment changes. A transthoracic echocardiogram showed no significant wall motion abnormalities and preserved left ventricular systolic function with an ejection fraction of 69%. There were no obvious findings that would suggest paradoxical or intracardiac embolism.

In the laboratory data, the high-sensitivity cardiac troponin-I level was 22 ng/L at arrival (upper reference limit of 34.2 ng/L for males, coefficient of variation of 3.5%, ARCHITECT i2000SR STAT hs-cTnI assay, Abbott Laboratories, North Chicago, IL, USA), but it reached up to 86 ng/L two hours later. The real-time polymerase chain reaction (PCR) test cycle threshold value of COVID-19 was 39.13 at admission. His C-reactive protein was increased to the level of 0.24 mg/dL, and his low-density lipoprotein cholesterol level was 100 mg/dL at arrival. Based on a diagnosis of non-ST-elevation acute myocardial infarction (NSTEMI) with Killip class I, he was administered 200 mg of aspirin and immediately transferred to our catheterization laboratory.

**Figure 1:**
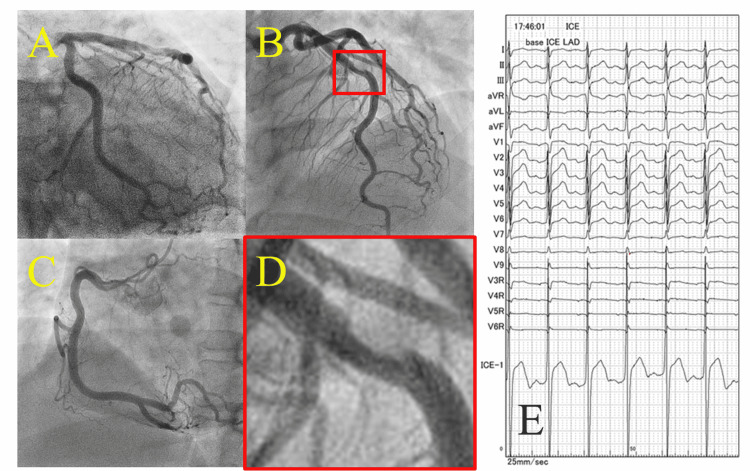
Pre-intervention CAG and electrocardiogram (A) and (B) Left coronary artery. (C) Right coronary artery. (D) Large-scale view of the culprit lesion in the middle segment of the LAD. (E) Body surface and intracoronary electrocardiogram. Despite no significant ST-segment changes in the body surface electrocardiogram, the intracoronary electrocardiogram of the LAD showed ST-segment elevation. CAG: Coronary angiogram; LAD: Left anterior descending artery.

An emergency coronary angiography (CAG) revealed 50% stenosis with thrombotic occlusion in the middle segment of the left anterior descending coronary artery (LAD) (Figure 1, Panels A-D), and primary percutaneous coronary intervention (PCI) was indicated. He was administered 20 mg of prasugrel. Intracoronary electrocardiogram of the LAD showed ST-segment elevation (Figure 1, Panel E).

**Figure 2:**
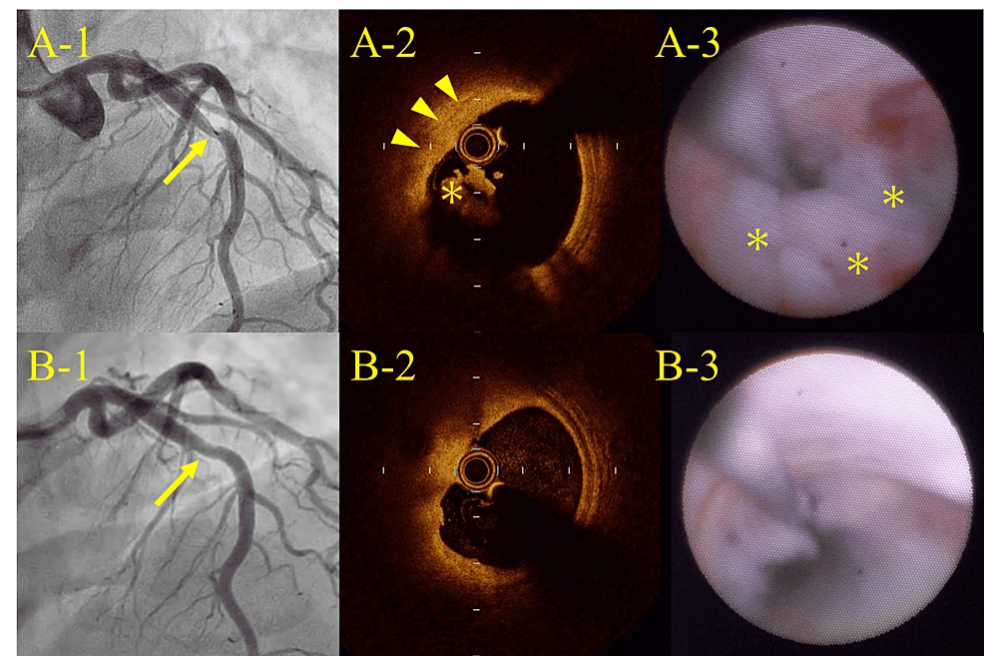
Pre-intervention (A) and post-intervention (B) images of CAG, OCT, and CAS Pre-intervention CAG (A-1) revealed 50% stenosis with a filling defect in the middle segment of the LAD. OCT (A-2) revealed the presence of plaque erosion (arrowhead) with mixed thrombus (asterisk) on a lipid-rich plaque. CAS (A-3) revealed the presence of a light-yellow plaque with mixed thrombus (asterisks). Post-intervention CAG (B-1) confirmed preserved TIMI 3 flow as seen at the beginning of the procedure. OCT (B-2) and CAS (B-3) revealed a significant reduction of intracoronary thrombus. CAG: Coronary angiogram; OCT: Optical coherence tomography; CAS: Coronary angioscopy; LAD: Left anterior descending artery; TIMI: Thrombolysis in myocardial infarction.

OCT and coronary angioscopy (CAS) revealed plaque erosion with mixed thrombus on a lipid-rich plaque (Figure 2, Panels A1-A-3). Aspiration thrombectomy was performed, but no apparent thrombus was aspirated. Plain old balloon angioplasty was performed using a 4.0-mm semi-compliant balloon with the nominal pressure of 8 atm, and TIMI (thrombolysis in myocardial infarction) flow grade 3 was maintained. OCT examination revealed a significant reduction of intracoronary thrombus and a minimal lumen area of 6.65 mm^2^. Taking all the data including symptom relief, angiographical appearance, OCT-derived residual lumen size, and residual thrombus volume (Figure 2, Panel B-2), we completed PCI without stent deployment. Intravenous heparin was administered for 48 hours after PCI, and aspirin was continued as a single antiplatelet therapy thereafter. Peak post-PCI CK and CK-MB levels reached 368 U/L and 31 U/L, respectively. The patient was discharged in a favorable clinical course seven days after PCI. He has been free from any cardiac events thereafter for six months.

## Discussion

COVID-19 became widely known to affect multiple organ systems with a broad spectrum of manifestations. COVID-19 infection also increases the risk of arterial and venous thrombotic complications [1]. Several reports suggest that the influence of COVID-19 infection on the hemostatic-thrombotic system may sustain for several weeks [2].

Although there are a number of reports regarding the association between COVID-19 infection and AMI, its exact mechanisms have not been completely elucidated. Endothelial injuries in patients with COVID-19 have been reported in pathological investigations [3], and SARS-CoV-2 attacks organs with high angiotensin-converting enzyme 2 (ACE2) expression such as endothelial cells [3]. Some studies indicated that the direct effect of SARS-CoV-2 on endothelial cells and the exaggerated inflammatory response as cytokine storm are likely to precipitate cardiovascular events through ACE2 receptor downregulation, platelet activation, hypercoagulability, and effects on endothelial cells including activation, injury, dysfunction, and apoptosis [4].

Herein, we reported a case of a patient with a NSTEMI who suffered from COVID-19 infection one month earlier. To the best of our knowledge, this is the first case report demonstrating the characteristics of culprit plaque of acute coronary thrombosis, which is most probably related to COVID-19 infection evaluated by multiple intracoronary imaging modalities. In the present case, the patient had several risk factors for coronary artery disease but no history of coronary artery disease or anginal symptoms. The OCT and CAS images revealed the presence of plaque erosion with mixed thrombus on a lipid-rich plaque without rupture or calcification.

Plaque erosion occurs without cap disruption where flowing blood comes into direct contact with the intimal surface lacking endothelial cells, and it is the second major mechanism of AMI [5]. The current OCT system cannot image endothelial cells despite its high resolution (≈10 µm). Therefore, the diagnosis of OCT-defined erosion rested primarily on the exclusion criterion of the absence of fibrous cap disruption [6]. Endothelial injuries caused by SARS-CoV-2 may expose the subendothelial layer and provide a thrombogenic status on the surface of coronary arteries, leading to the occurrence of AMI caused by plaque erosion. A recent case report suggested that plaque erosion might be a prevalent pathogenesis of myocardial infarction in COVID-19 patients [7]. The present case report supports this notion by OCT and CAS images and helps understand the underlying mechanisms of cardiac complications following COVID-19 infection.

## Conclusions

We conclude this case report by stating that endothelial injuries caused by SARS-CoV-2 may induce plaque erosion, leading to the occurrence of acute myocardial infarction that is complicated with COVID-19 infection.
